# Diagnostic and Prognostic Value of the Cerebrospinal Fluid Concentration of Immunoglobulin Free Light Chains in Clinically Isolated Syndrome with Conversion to Multiple Sclerosis

**DOI:** 10.1371/journal.pone.0143375

**Published:** 2015-11-25

**Authors:** Gleb Makshakov, Vladimir Nazarov, Olga Kochetova, Elena Surkova, Sergey Lapin, Evgeniy Evdoshenko

**Affiliations:** 1 Municipal Clinical Hospital No 31, City Center of Multiple Sclerosis and Autoimmune Diseases, Saint-Petersburg, Russian Federation; 2 First Pavlov State Saint-Petersburg Medical University, Center for Molecular Medicine, Laboratory of Autoimmune diagnostics, Saint-Petersburg, Russian Federation; Istanbul University, TURKEY

## Abstract

**Background and objective:**

In this study, we evaluated the diagnostic and prognostic significance of cerebrospinal fluid free light chains (CSF FLC) at the time of clinically isolated syndrome (CIS).

**Methods:**

We compared FLC-parameters at the moment of CIS in patients with conversion to multiple sclerosis (MS) after 2 years (CIS-MS), patients who remained stable both clinically and radiologically after 2 years (CIS-nonMS), patients with non-inflammatory neurologic diseases (NIND) as a comparison group and patients with other inflammatory neurologic diseases (IND) with intrathecal oligoclonal bands (OCB) synthesis. ROC-analysis was conducted to define FLC-assay characteristics and cut-off values. We also compared FLC-concentrations in CIS patients to determine their OCB-status. A correlation analysis was performed between FLC-concentrations and the expanded disability scale score (EDSS), annualized relapse rate (ARR) and MRI-activity (i.e., number of new and gadolinium-enhancing (Gd+) lesions) in patients.

**Results:**

The levels of kappa-FLC (k-FLC_*CSF*_) and lambda-FLC (λ-FLC_*CSF*_) as well as kappa- and lambda-quotients (Q-k and Q-λ) were elevated in CIS-MS compared to the CIS-nonMS and NIND groups. These levels did not differ significantly when compared with the IND group. We identified several patients with high k-FLC_*CSF*_ and λ-FLC_*CSF*_ in OCB-negative CIS and IND groups. The level of k-FLC_*CSF*_ production was significantly higher in OCB-positive patients in the CIS-MS group compared to the CIS-nonMS group. The concentrations of k-FLC_*CSF*_ and Q-k in the CIS-MS group showed significant correlation with the level of EDSS after 2 years (k-FLC: r = 0.4477,p = 0.0016; Q-k: r = 0.4621, p = 0.0016). λ-FLC_*CSF*_ and Q-λ inversely correlated with the number of Gd+ lesions (CSF λ-FLC: r = -0.3698, p = 0.0223; Q-λ: r = -0.4527, p = 0.0056).

**Conclusion:**

The concentration of CSF FLC predicts conversion to MS within 2 years following CIS. OCB-positive patients with an early conversion have a higher concentration of CSF-FLC. We have also shown a prognostic significance of k-FLC_*CSF*_ for future EDSS-progression.

## Introduction

Multiple sclerosis (MS) is a debilitating neurologic immune-mediated disease. In the majority of patients, the condition begins as a single clinical episode, called clinically isolated syndrome (CIS). Conversion from CIS to clinically definite MS (CDMS) is a frequent, but not universal, occurrence. Indeed, a significant amount of those who have experienced the typical symptoms of CIS do not develop CDMS. Potential prognostic biomarkers of conversion to CDMS could increase the clinical vigilance of MS and help define a potential therapeutic approach for patients with CIS.

Increased intrathecal immunoglobulin (Ig) production and clonal restriction that result in the formation of IgG oligoclonal bands (OCB) in the cerebrospinal fluid (CSF) are well described in both CIS and MS [1,2]. Of all tests, the OCB IgG is recognized as a “gold standard” for laboratory diagnostic of MS, although it is not ideal because of the moderate specificity for CDMS and some potential for subjective interpretation [3]. It has been shown that OCB detection at the time of CIS can predict the transformation to clinically definite MS [4]; however, this quantitative method cannot predict the rate of conversion and the range of disability among different patients with positive OCB-status.

Several studies have noted a persistently increased production of CSF immunoglobulin free light chains (FLC) kappa and lambda (k-FLC, λ-FLC, respectively) in CIS and MS [5–7]. It has been shown that the high concentration of k-FLC_*CSF*_ can reliably predict the conversion from CIS to MS [8]. A correlation between the concentrations of CSF FLC and disability outcomes in CIS and MS remains unresolved [9–11]. Increased intrathecal synthesis of FLC was detected in a small subpopulation of OCB-negative patients [3,12,13].

FLC in CSF can be measured with an enzyme-linked immunosorbent assay (ELISA) with a sufficiently high sensitivity and specificity for the MS diagnosis [3]. The quantitative nature of ELISA is an advantage because a concentration of FLC could reflect the difference in the amount of inflammation in the CNS of patients who have the same OCB-status, which is of practical significance.

In this study we aimed 1) to investigate the significance of FLC in the diagnosis of MS at the time of CIS; 2) to evaluate a prognostic significance of FLC CSF concentrations that were obtained at CIS in patients who converted to CDMS after 2 years; and 3) to assess the relevance of FLC detection in combination with OCB as an additional diagnostic tool for MS diagnostics, particularly in OCB-negative patients with high concentrations of FLC in the CSF.

## Material and Methods

### Subject selection

This study was approved by the local Ethics Committee of the Municipal Clinical Hospital No 31, City Center of Multiple Sclerosis and Autoimmune Diseases. All participants provided written informed consent.

In this study, we retrospectively analyzed the data of paired serum and CSF samples that were collected from patients with CIS that routinely underwent a lumbar puncture between 2012 and 2015. We reviewed the following patient groups: patients with CIS, who converted to MS within 2 years after their first relapse (CIS-MS group), n = 98; patients with CIS, who did not convert to MS within 2 years of the observation (CIS-nonMS), n = 41; a comparison group of patients with non-inflammatory neurologic diseases (NIND), n = 43; and patients with other inflammatory neurologic diseases (IND) with defined intrathecal oligoclonal immunoglobulin production in most cases, n = 16 (Table 1). OCB-status and FLC parameters in the CIS groups were measured during the time after their first attack. Patients from the NIND and IND groups were admitted to our clinic with suspicion of probable MS. All patients had multifocal MRI lesions, but further evaluation ruled out MS. In the IND group, other inflammatory or autoimmune disorders were revealed.

**Table 1:** General characteristics of CIS-MS, CIS-nonMS, NIND and IND groups.

Variable	CIS-MS (n = 98) (n (%))	CIS-nonMS (n = 41) (n (%))	Group of comparison (NIND) (n = 43)	IND (n = 16) (n (%))
Age at Onset (Mean ± St. Dev)	32 (±9.6)****	33,0 (±11.5)*	40.5 (±13.4)†	33 (±17.3)†
Sex (F)	67 (68)	33 (81)	33 (77)	12 (75)
OCB (Positive)	91 (93)	20 (49)	0 (0)	13 (81)
Spectrum of disorders in the NIND and IND groups:			Adult-onset ataxia (n = 1), autonomic system disorder (n = 4), B12-deficiency (n = 1), hepatitis C (n = 1), intoxication, pontine myelinolysis suspected (n = 1), schizophrenia (n = 1), seizure disorder (n = 2), tension headache (n = 2), thyreotoxicosis (n = 1), traumatic brain injury (n = 2), miscellaneous (n = 12), vascular (n = 14), vertigo (n = 1)	CNS tuberculosis (n = 1), vasculitis (n = 3), CNS toxoplasmosis (n = 1), NeuroAIDS (n = 1), PML/ AIDS (n = 1), Vogt-Koyanagi-Harada syndrome (n = 1), primary lateral sclerosis (n = 1), paraneoplastic encephalitis (n = 2), Sjogren’s syndrome (n = 1), Neurosarcoidosis (n = 2), ADEM (n = 1), isolated CNS vasculitis (n = 1)

CIS-MS, patients who converted to multiple sclerosis within 2 years under observation; CIS-nonMS, patients who did not convert to multiple sclerosis within 2 years under observation; NIND, non-inflammatory neurologic diseases; IND, inflammatory neurologic diseases; AIDS, acquired immunodeficiency syndrome; PML, progressive multifocal leucoencephalopathy; OCB, oligoclonal bands.

^**†**^ for NIND and IND groups the age is counted at the moment of lumbar puncture.

**** p-value <0.0001 for comparison of age between CIS-MS and NIND groups;

* p-value <0.05 for comparison of age between the CIS-nonMS and NIND group.

### Prognostic factors

EDSS values were calculated for the CIS-MS group after 2 years together with the relapse rate. MRI-measures were assessed only in patients who performed MRI regularly at least once a year since the time of the first symptoms.

### OCB and FLC detection

Serum samples were collected at the date of lumbar puncture. OCBs were detected using a routine agarose gel isoelectrofocusing (IEF) with subsequent blotting and immunostaining. Analysis of the results was conducted based on international recommendations [14].

FLC-concentrations were measured using a novel ELISA assay (Polignost Ltd., St. Petersburg, Russia) based on monoclonal anti-k and anti-λ antibodies directed against cryptic epitopes of free FLC molecules. During the standardization of FLC measurement techniques, we compared the immunonephelometric assay to the ELISA, which produced better results. To interpret the concentrations of FLC in CSF regarding their serum concentration, we calculated FLC quotients (Q-k and Q-λ, respectively): Q-FLC = FLC_*CSF*_ / FLC_*SERUM*_.

Albumin concentrations were counted both in the serum and CSF in CIS-MS and NIND groups using nephelometry to evaluate the integrity of the blood-brain barrier and to exclude possible leakage of FLC from the blood into the CSF. The albumin CSF/serum ratio was calculated, and FLC indices were defined as ratios of Q-FLC/Qalb (I-k and I-λ, respectively) to assess the influence of potential blood brain barrier disruption on CSF concentrations of FLC.

### Statistical analysis

Data were processed with GraphPad Prism 6 (GraphPad Software Inc., CA, USA). To test for normality, the Kolmogorov-Smirnov test was applied. All subject groups demonstrated a non-normal distribution. Comparisons between groups were made using non-parametric Mann-Whitney t-test. A two-sided p-value < 0.05 was considered to indicate statistical significance. We used receiver-operator curve (ROC) analysis to evaluate diagnostic sensitivity and specificity of our FLC-assay in the CIS-MS group with the NIND group as a control and to calculate cut-off values for each parameter. The analysis of correlation was performed using non-parametric Spearman’s r-value.

## Results

In a cohort of NIND patients, the mean age at the time of lumbar puncture was significantly higher than in the CIS groups. OCB predominance was the highest in the CIS-MS group (93%) compared to the CIS-nonMS (49%, p-value <0.0001, Chi-square test) or IND (81%, p-value = 0.1458, Chi-square test).

**Fig 1:**
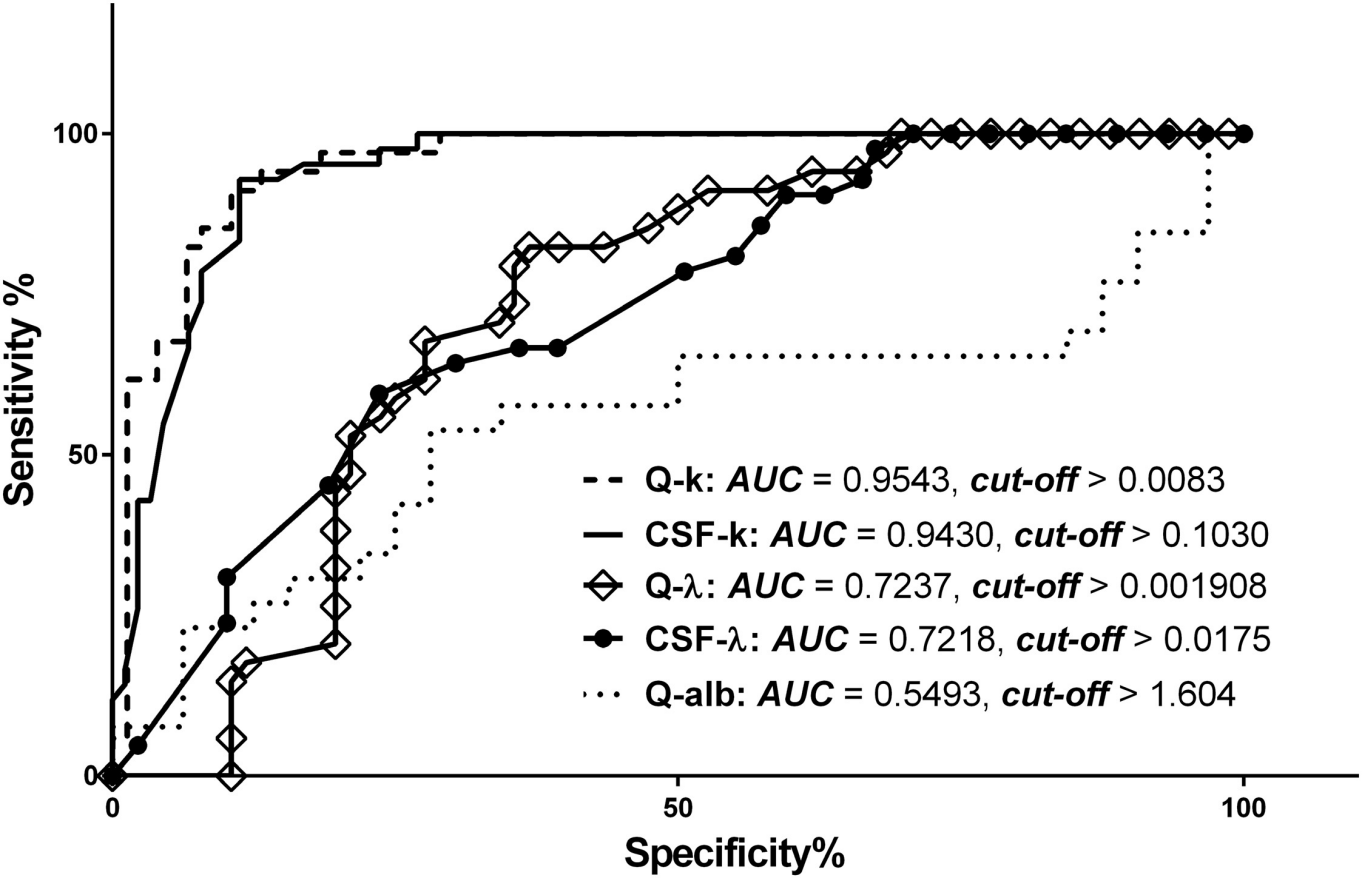
Receiver operator curve (ROC) characteristic analysis between CIS-MS and control group. AUC: area under curve; CSF k-FLC: concentration of kappa free light chains in cerebrospinal fluid (CSF); CSF λ-FLC: concentration of lambda free light chains in CSF; Q-k: CSF/serum quotient of concentrations of kappa free light chains; Q-λ: CSF/serum quotient of concentrations of lambda free light chains.

In our study, k-FLC_*CSF*_ and Q-k were shown to be markers with the best diagnostic characteristics for patients with CIS compared with λ-FLC_*CSF*_ and Q-λ, according to ROC-analysis between the CIS-MS and NIND groups (Fig 1). The summary is presented in Table 2.

**Table 2:** Receiver operator characteristic analysis of CSF free light chain (FLC) levels.

	Sensitivity (%)	Specificity (%)	AUC	Likelihood ratio	Cut-off (mcg/ml (for FLC))
**CSF-k**	92.9	88.8	0.9430	8.264	>0.1030
**Q-k**	88.24	89.5	0.9543	8.382	>0.0083
**CSF-λ**	31.0	89.9	0.7218	3.061	>0.0175
**Q-λ**	20.6	88.2	0.7237	1.739	>0.0019
**I-k**	40.0	92.31	0.7677	5.2	>0.0032
**I-λ**	4.0	92.31	0.5677	0.52	>0.00029
**Qalb**	23.1	93.75	0.5493	3.692	>1.604

AUC: area under the curve; CSF-k: concentration of kappa free light chains in cerebrospinal fluid (CSF); CSF-λ: concentration of lambda free light chains in CSF; Q-k: quotient of concentrations of kappa free light chains; Q-λ: quotient of concentrations of lambda free light chains; I-k: Q-k/Qalb ratio; I-λ: Q-λ/Qalb ratio; Q-alb: quotient of CSF/serum albumin concentrations.

The CSF/serum albumin quotient (Qalb) was assessed to account for the potential endothelial barrier disruption. The results of the ROC-analysis showed that the CSF concentration of light chains was independent of endothelial barrier integrity because the Q-alb had the smallest area under curve (AUC) among all measures (Fig 1, Table 2). The ratios of the light chains quotients to Qalb (I-k, I-λ) demonstrated worse test characteristics than those counted solely for Q-k and Q-λ.

**Fig 2:**
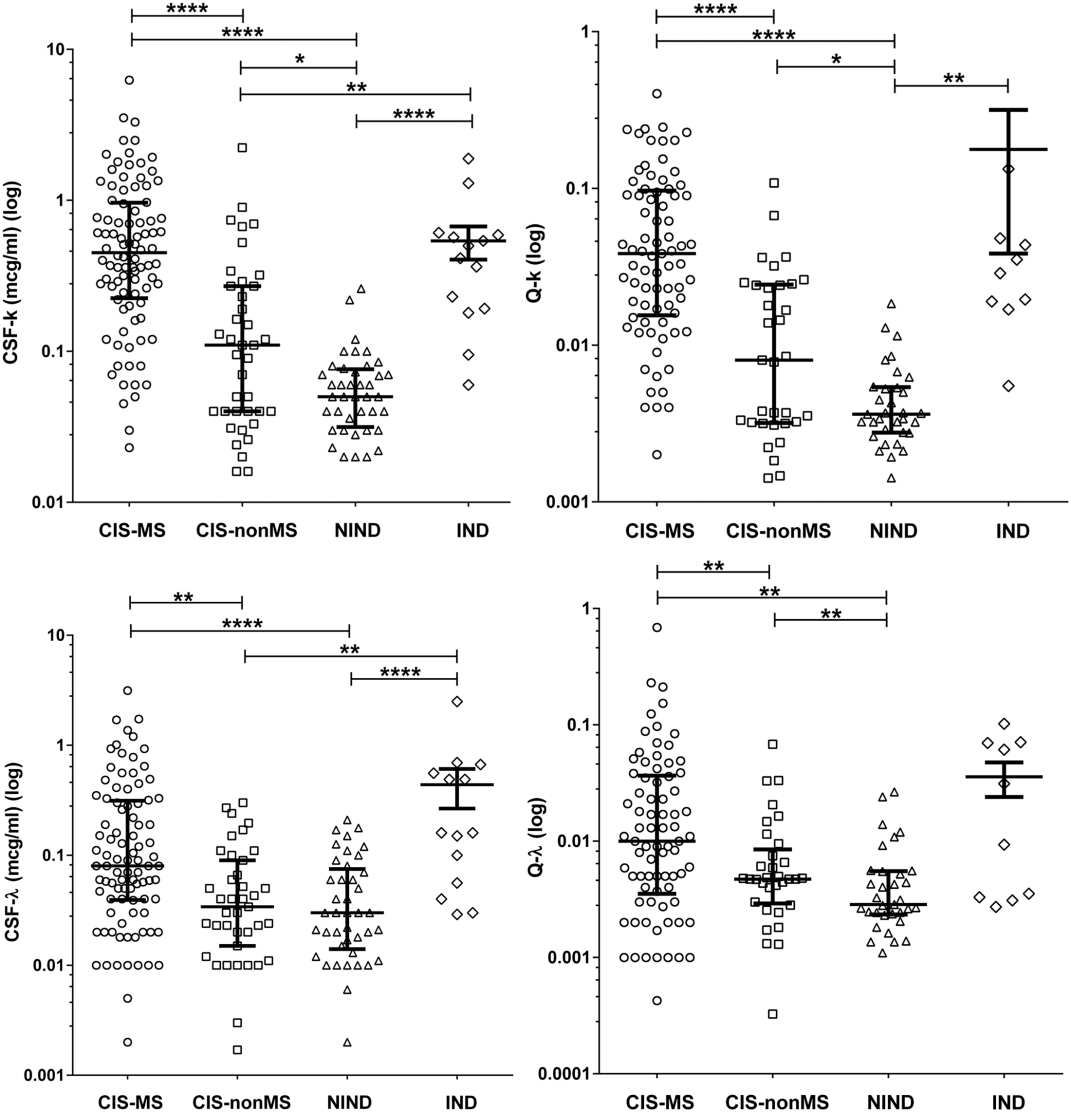
Levels of intrathecal FLC in different groups. CIS-MS: patients with clinically isolated syndrome that converted to multiple sclerosis after 2 years. CIS-nonMS: patients with clinically isolated syndrome that did not convert to multiple sclerosis after 2 years. NIND: patients with non-inflammatory neurologic diseases; ****−p<0.00001; **−p<0.01; *−p<0.05. Please also refer to Table 2 for more details.

The levels of k-FLC_*CSF*_ and λ-FLC_*CSF*_, as well as Q-k and Q-λ were elevated in both CIS-groups compared to the NIND-group. Patients in the CIS-MS group showed significant differences in the value of k-FLC_*CSF*_, λ-FLC_*CSF*_, Q-k and Q-λ in comparison with the CIS-nonMS group. k-FLC_*CSF*_ and Q-k values were significantly higher in the CIS-nonMS group compared to the NIND group, but the λ-FLC_*CSF*_ and Q-λ concentrations were not at a statistically significant level (Table 3, Fig 2). The levels of the FLC in other inflammatory disease groups demonstrated a high variety and did not significantly differ from the CIS-MS group, although this group showed a significant increase in k-FLC_*CSF*_, λ-FLC_*CSF*_, and Q-k values when compared to the CIS-nonMS group (Fig 2). The levels of λ-FLC_*SERUM*_ were also significantly increased when compared to the CIS-nonMS group (p = 0.0242, data not shown). Clearly, the IND group significantly differed from the NIND group in all measures (Fig 2)

**Table 3:** Concentrations of free light chains in CSF and serum and t-test results (data shown as median and IQR).

	CIS-MS (mcg/ml)	CIS-nonMS (mcg/ml)	NIND (mcg/ml)	IND (MCG/ML)
**CSF-k**	0.45 (0.225–0.965)	0.11 (0.004–0.27)	0.05 (0.032–0.076)	0.45 (0.189–0.595)
**CSF-Λ**	0.08 (0.039–0.313)	0.034 (0.015–0.09)	0.03 (0.014–0.075)	0.16 (0.052–0.585)
**Q-k**	0.039 (0.016–0.099)	0.008 (0.003–0.024)	0.004 (0.003–0.005)	0.032 (0.019–0.069)
**Q-Λ**	0.01 (0.0035–0.037)	0.005 (0.003–0.008)	0.003 (0.002–0.006)	0.02 (0.003–0.07)

CSF-k: concentration of k-FLC in CSF; CSF-λ: concentration of λ-FLC in CSF; Q-k: the ratio of concentrations of kappa FLC in CSF/kappa FLC in serum; Q-λ: the ratio of concentrations of lambda FLC in CSF/lambda FLC in serum; NIND: non-inflammatory neurologic disorders; IND: other inflammatory neurologic diseases.

**Fig 3:**
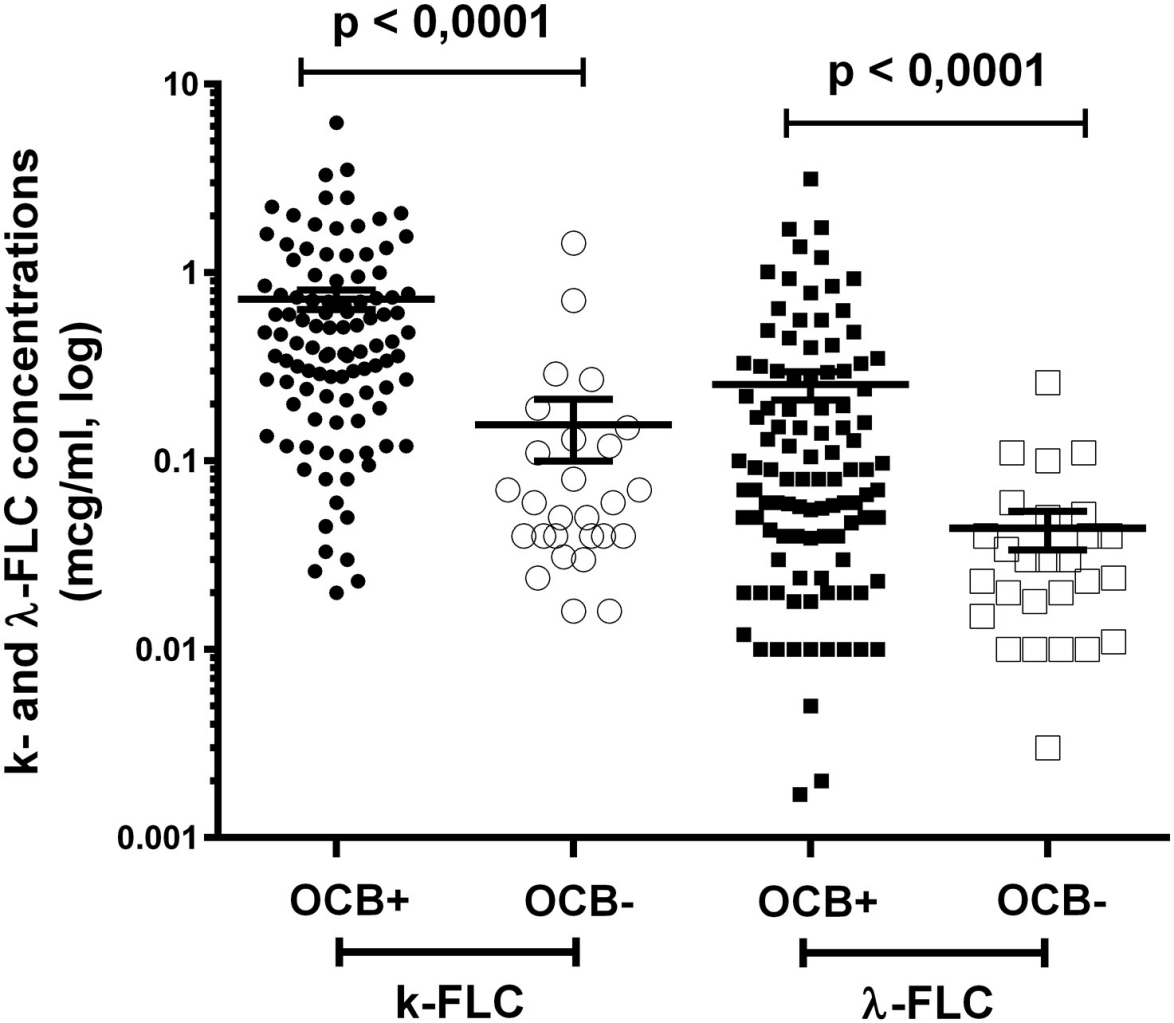
Concentration of k-FLC_*CSF*_ and λ-FLC_*CSF*_ in OCB-positive and OCB-negative patients in both CIS-MS and CIS-nonMS groups (data coupled). CIS-MS: patients with clinically isolated syndrome who converted to multiple sclerosis after 2 years; CIS-nonMS: patients with clinically isolated syndrome who did not convert to multiple sclerosis after 2 years; OCB: oligoclonal bands.

We evaluated the predominance of OCB-positive patients in both CIS-MS and CIS-nonMS groups. The number of OCB-positive patients was significantly higher in the CIS-MS group than in the CIS-nonMS group (n = 91 (93%); n = 20 (49%), respectively, p-value <0.0001), as shown in Table 1. No samples were positive for OCB in the NIND group (Table 1). It is possible that all patients who presented with CIS can then develop CDMS, so the detection of intrathecal immunoglobulin production is of primary importance for assessing the prognosis of such patients. To assess the relationship between OCB and FLC production, we compared FLC levels in OCB-positive and OCB-negative patients in both CIS-MS and CIS-nonMS groups. The concentrations of both k-FLC_*CSF*_ and λ-FLC_*CSF*_ were significantly increased in OCB-positive patients, (p<0.0001) as shown in Fig 3.

**Fig 4:**
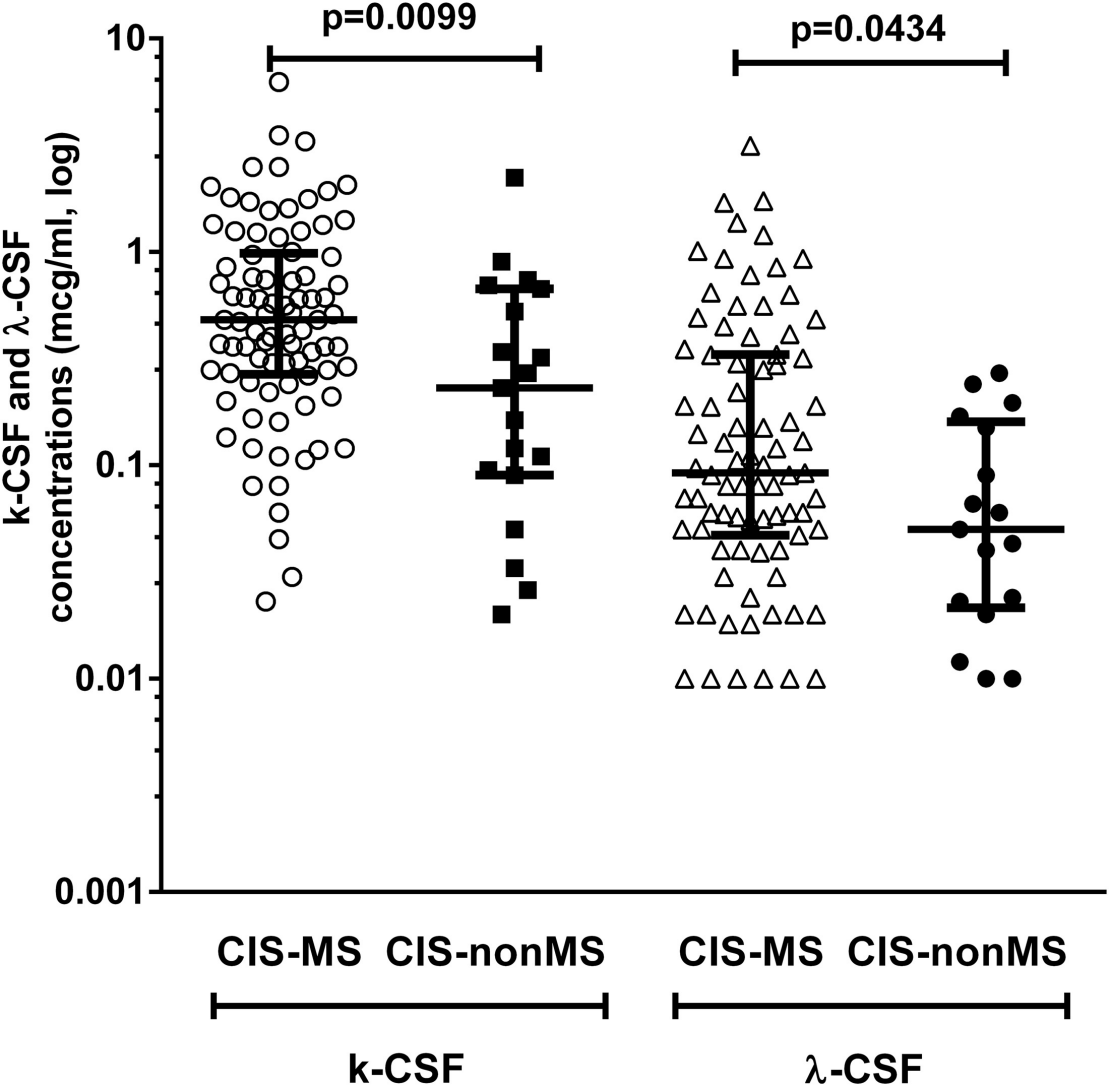
Concentrations of k-FLC_*CSF*_ and λ-FLC_*CSF*_ in OCB-positive patients in CIS-MS and CIS-nonMS groups. CIS-MS: patients with clinically isolated syndrome who converted to multiple sclerosis after 2 years; CIS-nonMS: patients with clinically isolated syndrome who did not convert to multiple sclerosis after 2 years; OCB: oligoclonal bands.

To detect the difference in FLC production between OCB-positive early converters and non-converters, we compared concentrations of k-FLC_*CSF*_ and λ-FLC_*CSF*_ between the CIS-MS and CIS-nonMS groups. The concentrations of both k-FLC_*CSF*_ and λ-FLC_*CSF*_ were significantly higher in the CIS-MS group than in the CIS-nonMS group (p = 0.0099 and p = 0.0434 for k-FLC_*CSF*_ and λ-FLC_*CSF*_, respectively; Fig 4).

The concentration of k-FLC_*CSF*_ and Q-k in the CIS-MS group showed a significant correlation with the level of EDSS progression after two years (n = 40, k-FLC: r = 0,4184,p = 0.0072; Q-k: r = 0.4101,p = 0.0117) but failed to show a statistically significant correlation with relapse rate, time till second relapse or the number of new T2- and Gd+ lesions. λ-FLC_*CSF*_ and Q-λ were inversely correlated with the number of Gd+ lesions (n = 34, CSF λ-FLC: r = -0.4311, p = 0.0109; Q-λ: r = -0.4933, p = 0.0041) but did not show a significant level of correlation with EDSS, relapse rate, time till second relapse or MRI measures.

In the CIS-MS group, 29% (n = 2/7) of OCB-negative patients were shown to produce elevated amounts of k-FLC_*CSF*_. This was also true for 33% (n = 7/21) of OCB-negative patients in the CIS-nonMS group, although the level of their production was lower than in the CIS-MS group (CIS-MS: median = 1.07 (0.71–1.43); CIS-nonMS: median = 0.15 (0.12–0.27). The abnormal synthesis of k-FLC_*CSF*_ was also detected in 66% (n = 2/3) of OCB-negative IND patients (median = 0.134 (0.069–0.57). We could not statistically compare concentrations due to small sample sizes. Analysis of λ-FLC concentrations revealed an increased production of λ-FLC_*CSF*_ in 86% of patients (n = 6/7, median = 0.035 (0.018–0.11)) in the CIS-MS group and 71% in the CIS-nonMS group (n = 15/21, median = 0.034 (0.023–0.052)). No significant difference was found between these groups (t-test, p = 0.8918). In the IND group, 3/3 patients were found to produce higher concentrations of -FLC_*CSF*_ than a settled cut-off (median = 0.134 (0.069–0.57)).

## Discussion

The differential diagnosis of MS and the prediction of the disease course require a complex approach, including the use of reliable and robust laboratory assays. While the detection of OCB IgG is considered a “gold standard” for MS diagnostics, it possesses some drawbacks because of its qualitative nature. Quantitative assessment of CSF FLC is considered by some authors to be a promising additional test for MS diagnosis [15].

The goal of this research was to evaluate the diagnostic and prognostic significance of CSF FLC in CIS with conversion to CDMS in 2 years at follow-up.

The analysis of OCB-prevalence in our population revealed a 93%-positivity for OCB in the CIS-MS group, which aligns with the known data of OCB sensitivity [1, 4, 6]. Our ROC-analysis revealed that the k-FLC_*CSF*_ concentration and Q-k had the best characteristics based on AUC compared with other parameters, which is in line with other studies [3,15] (Fig 1). The sensitivity of k-FLC_*CSF*_ detection was nearly similar to that of OCB detection. Blood-brain barrier functions were not shown to improve the results of the test, as measured by Qalb, I-k and I-λ (Fig 1, Table 2); however, opposing results were shown in a number of studies by other authors [5–6]. Our data indicate that FLC is, above all, a product of intrathecal inflammation.

The levels of FLC production in both CIS groups were higher than in the NIND group, which reflected the presence of an intrathecal inflammation process in patients in the CIS groups, which is characteristic of MS (Table 2, Fig 2). These data are in line with other studies of FLC diagnostic significance in CIS and MS [5,7]. We also found that patients in the CIS-MS group had significantly higher levels of FLC production than in the CIS-nonMS group (Table 2, Fig 2A–2D). This can be explained by more pronounced inflammatory changes in patients with early conversion to MS that can also be supported by a higher proportion of OCB-positive patients in the CIS-MS group than in the CIS-nonMS group (Table 1). It is evident that the OCB-status has a profound impact on the values of the FLC-parameters, which is supported by results of the comparison of FLC-production in the OCB-positive versus OCB-negative CIS patients (Fig 3). For this reason, we tested the difference in FLC production between OCB-positive patients in CIS-MS and CIS-nonMS groups. We showed that OCB-positive CIS-MS patients have significantly higher levels of k-FLC_*CSF*_ and λ-FLC_*CSF*_ than did CIS-nonMS patients (Fig 4). Hence the difference in time until CDMS could be better stratified using the level of CSF FLC when compared to OCB. In this study, we also compared CIS groups with a group of other inflammatory diseases, which mostly tested positive for intrathecal OCB synthesis. The levels of FLC production in this group showed a high variety within subjects that mainly depended on pathological processes in each case. The CIS-nonMS group showed significantly lower levels of FLC that also reflected a lower range of intrathecal inflammation compared to the IND group, whereas no significant difference was observed between the CIS-MS and IND groups. A serious limitation of this study was a small sample size because our center is specialized overall for patients with MS. While our data agree with results of larger cohorts [5,11], some studies demonstrated that k-FLC detection was more specific for MS than for inflammatory diseases [7]. Quantitative FLC detection reflects only the amount of inflammation but does not reveal the nature of the humoral response and the number of antibody-producing clones and cannot be used for differential diagnosis.

We documented OCB-negative patients with an increased production of k-FLC_*CSF*_ in both CIS groups and the IND group, but we were unable to statistically compare concentrations due to a small number of samples. Similar observations of k-FLC_*CSF*_ levels have been previously reported in a number of studies [3, 12, 13]. Increased production was also detected for λ-FLC_*CSF*_ in OCB-negative CIS patients. No difference in λ-FLC_*CSF*_ concentration was detected for groups with and without early conversion to MS. To our knowledge, no such data exists for λ-FLC_*CSF*_ measurements. These findings might reflect the stage of MS development wherein there are apparent signs of inflammation but no obvious OCB production, or they may indicate a different phenotype of the patients. More extensive research on this issue was beyond the scope of this article. It is probable that a detection of increased FLC production in OCB-negative patients could provide more information concerning the amount of intrathecal inflammation, thereby being beneficial for MS diagnosis. A combination of both methods could increase a diagnostic sensitivity for the potential conversion to MS at the time of CIS. Studies in a larger cohort of patients will be needed to define this.

We performed a correlation analysis to determine the prognostic significance of FLC. We showed a strong correlation between the amount of CSF k-FLC and EDSS after two years in the CIS-MS group, although other markers of disease activity failed to show such a level of significance. Previous studies have failed to show an association between FLC concentration and EDSS level and relapse rates in CIS that converted to CDMS [11]. These data are supported by earlier studies showing a connection between high levels of CSF FLC production and disability outcomes in patients with MS [9, 10]. The existence of a significant correlation with short-term disability but not with relapse-rate or time to second relapse, is interesting because these markers of disease activity are considered to be connected. The detection of high amounts of FLC could indicate a higher risk for disability progression for a distinct patient starting at the moment of MS diagnosis because intrathecal immunoglobulin and FLC synthesis was shown to be relatively stable over time [16]. It remains to be evaluated in bigger cohorts of patients with longer follow-up period whether free light chains are a putative prognostic marker for a long-term disability progression. The current study has limitations because it was retrospective and was based on examinations performed by several different neurologists.

The level of λ-FLC_*CSF*_, but not the level of k-FLC_*CSF*_, inversely correlated with the amount of Gd+ lesions. To our knowledge, this was not shown before. One can assume that MS patients may produce different radiology measurements based on kappa/lambda production intensity. In a study by Rudick et al., k-FLC_*CSF*_ showed a weak but significant correlation with baseline Gd+ lesions and T2-lesions amount [16] that was not demonstrated in our study. The limitation of this study was the assessment of MRI in routine clinical practice without a unified protocol and in the absence of an independent viewer, and therefore these findings require further investigation.

Thus, the increased production of immunoglobulin kappa-free light chains was significantly higher in patients who converted to CDMS during 2 years under observation. It was shown that the amount of synthesis between CIS groups was different in OCB-positive patients. These facts could make an impact on therapeutic strategies because the early detection of patients who will convert to CDMS at the moment of CIS may improve their outcomes. Whether the detection of increased synthesis of FLC in OCB-negative patients differs between early and late converters to CDMS and whether this analysis has some practical implication in these patients needs to be evaluated in further studies. We also demonstrated the prognostic significance of kappa and lambda FLC at the time of CIS in patients who converted to MS within 2 years.

## Conclusion

The concentration of CSF FLC predict conversion to MS within two years after CIS. k-FLC_*CSF*_ and Q-k values have the best diagnostic characteristics compared to λ-FLC_*CSF*_ and Q-λ values. OCB-positive patients with early conversion have higher concentrations of CSF FLC. Increased FLC production was found in OCB-negative patients mainly of λ-subtype. k-FLC_*CSF*_ concentrations were shown to have prognostic significance for future disability after 2 years.
